# Chloroform in Dentistry

**Published:** 1848-02

**Authors:** George Buchanan


*Chloroform in Dentistry.* By George Buchanan.—The following case may prove interesting, as proving one of the many advantages the new anæsthetic agent, chloroform, possesses over ether:—

A married lady, twenty-six years of age, applied to me in the beginning of August, to have several teeth extracted; I administered ether—by a modification of Hooper's Inhaler—but the effects produced were so violent as to preclude all possibility of operating. A week afterwards I again attempted to narcotize her, thinking that the previous excitement might have been caused by the great dread she had of the ether—which fears were now removed,—but on this occasion the effects were still more violent. After the lapse of ten days she again applied to me, and at her urgent request I made a third attempt, which was followed with similar results to the two former. On each occasion the patient suffered severely from headach and sickness, for some hours after the application of the ether.

Thinking this a favourable case to test the efficacy of chloroform, on the 19th instant I persuaded the lady to allow me to give it a trial. I put half a drachm on a sponge, holding it to the mouth and nose; in half a minute the patient was sound asleep, but I continued the application of the sponge for a minute; I then extracted two superior molars, cut across and destroyed the nerves of the four upper incisors: this operation occupied about three minutes, during which time the patient was in a sound sleep, and on recovering, which she did in a few minutes after the operation was over, she said she had had a pleasant dream, felt no pain, and was quite unconscious of what had been done. No headach, or any disagreeable after effects.—*London Lancet.*

